# Standardizing patient-reported outcomes across diseases: development of a novel generic patient-reported outcome set

**DOI:** 10.3389/frhs.2025.1497055

**Published:** 2025-10-02

**Authors:** Preston Long, Alize Rogge, Ann-Kristin Porth, Evelyn Gross, Liselotte Fierens, Belle H. de Rooij, Nadia Kamminga, Tanja Stamm

**Affiliations:** 1 Institute for Outcomes Research, Medical University of Vienna, Vienna, Austria; 2 Medizinische Klinik m. S. Psychosomatik, Charité University Medicine Berlin, Berlin, Germany; 3 Department of Medicine III, Division of Endocrinology and Metabolism, Medical University of Vienna, Vienna, Austria; 4 Österreichische Morbus Crohn/Colitis ulcerosa Vereinigung (ÖMCCV), Vienna, Austria; 5 Department of Chronic Diseases and Metabolism, KU Leuven, Leuven, Belgium; 6 Research & Development, Netherlands Comprehensive Cancer Organisation, Utrecht, Netherlands; 7 Erasmus MC Cancer Institute, University Medical Center Rotterdam, Rotterdam, Netherlands; 8 Ludwig Boltzmann Institute for Arthritis and Rehabilitation, Vienna, Austria

**Keywords:** quality of life, patient-reported outcome measures, wellbeing, generic outcomes, H2O project

## Abstract

**Objectives:**

Patient-reported outcomes (PROs) are an essential component in the implementation of value-based health care. Up to now, no consensus exists on the appropriateness of PROs used across diseases, e.g., to allow for comparability or to assess disease impact. The aim of this study was to develop an international, multi-stakeholder consensus on a generic PRO set applicable for different stakeholders and diseases within of the Health Outcomes Observatory (H2O) project funded by the EU Innovative Medicines Initiative.

**Methods:**

To begin, a literature review was conducted to identify the most frequently utilized generic PROs followed by a three-round Delphi consensus procedure. The resulting outcome set was then cross-referenced with disease-specific outcome sets for lung and metastatic breast cancer, diabetes, and inflammatory bowel diseases to identify overlaps and gaps. Lastly, the identified generic outcome domains were mapped to the Max Neef's human needs model to explore the degree to which the generic domains address a general concept of wellbeing.

**Results:**

The literature search resulted in 2357 articles from which 190 PROMs and their measured domains were extracted. The Delphi consensus procedure reduced these to 10 core domains (mental, physical and social wellbeing, overall health status, fatigue, pain, sleep quality, sexuality, self-efficacy, treatment satisfaction). In comparison to the human needs model, needs such as identity and leisure were disregarded.

**Conclusions:**

The H2O generic outcome set presents a disease-generic, domain-centered PRO framework building the groundwork for health data spaces and supporting consistency in treatment outcomes across different sites, settings, and patient populations.

## Highlights

Generic outcomes sets can help complete patient profiles by ensuring that primary facets of human wellbeing are not overlooked due to focus on a specific disease. This paper explores the overlaps and gaps of current generic outcome sets in use compared to four disease specific outcome sets, leading to the development of a novel core outcome set. A scoping review, multi-stakeholder Delphi study, and mapping exercise were conducted for this purpose. Findings show that certain symptoms, such as fatigue and pain are standardly considered across disease areas. However, some external factors such as social and financial resource availability - key components of generic human wellbeing - are rarely considered. Further exploration highlights the strengths and limitations of a generic set value compared to disease-specific.

## Introduction

In recent years, information reported directly by patients about their health as well as their treatment processes and outcomes has become extremely valuable for informed decision-making in clinical settings, action-taking in regulatory services, quality assessment and research (1, 2). Patient-reported outcomes (PROs) and their respective measures (PROMs) assess subjective health information based on patients' perspective without further interpretation by third parties, e.g., by healthcare providers (3). With a growing body of PROMs in clinical trials, many initiatives such as the International Consortium for Health Outcomes Measurement (ICHOM) aim to standardize disease outcome sets to facilitate data interpretation on a large scale. Moreover, for many disease areas, no clear recommendations of the appropriate selection and use of PROMs can be considered the gold standard (4), i.e., overwhelmingly agreed upon as most valid and reliable, instead generic outcome sets are put in place.

PROs and their measures can be categorized on a generic, disease-unspecific level (e.g., SF-36 – Short form health survey), or on a disease-specific level (e.g., ADDQOL – Audit of diabetes-depended quality of life) (5). There is some evidence to suggest that generic outcomes, such as disease-unspecific health-related quality of life, are significantly correlated to the disease-specific counterpart; e.g., cancer-specific quality of life (6). Although, the degree of overlap and most appropriate contexts to use each remain vague.

Most generic PROMs offer assessment through several domains generating one profile of scores as part of results (5). This is especially interesting when comparing different disease populations to maximize value in treatments and to assess overall disease burden. Moreover, most generic PROMs have shown a growing body of evidence on its validity. However, when used as static measures for repeated measurements, its unspecific core often leads to difficulties in making changes in treatment visible; e.g., due to floor and ceiling effects (7). Moreover, across different generic health models, e.g., PROMIS-29 (8) and EORTC QLQ-C30 (9), inconsistencies of generic domains exist, raising the question of what the construct of well-being or overall health consists. Therefore, there is a need for a generic outcome set that explicitly captures the primary factors associated with wellbeing, and furthermore, such an outcome set is developed along with all relevant stakeholders from across multiple cultures. In this study, we aim to conduct a literature search to inform and execute a three-round Delphi consensus procedure (10) in an international and diverse group of stakeholders to establish a generic core outcome set. Then, the generic outcome set developed will be mapped to the Max-Neef's needs (11) model to assess overlaps of the underlying overall health constructs. By taking a holistic viewpoint, we aim to provide a generic outcome set usable largely irrespective of disease to maximize value from data gained from patients. This study is part of the Health Outcomes Observatory (H2O) project (https://health-outcomes-observatory.eu/). H2O aims to enable a value-based approach in healthcare systems, to improve sustainability by supporting optimized care delivery and the use of resources around outcomes that matter to patients (1).

## Methods

### Study design

This study consisted of five distinct methodological components:

1. Scoping literature review: The first step involved conducting a literature review to assess current generic PRO outcomes sets in practice and to identify their common components; the guiding research question was as follows: what validated and multistakeholder informed generic PRO sets have already been developed? Delphi Consensus Procedure: Findings from the review were prepared to set the background for an international, multi-stakeholder Delphi exercise in which a new, pragmatic generic PRO outcomes set was developed and customized for the needs of the H2O project;
2. H2O mapping exercise: the resulting generic core outcome set was then compared to diseases-specific core outcome sets developed for four disease areas in the H2O project: diabetes (12), lung cancer (13), metastatic breast cancer (14), and inflammatory bowel disease (15). The overlaps and divides between the four diseases and the generic outcome domains were explored. Then, the H2O findings were compared to two existing and well-established generic outcome frameworks: PROMIS (generic domain framework across diseases) (8) and the EORTC (generic domain framework across different cancer entities) (16);
3. Human Needs Exploration following Max Neef's theory of needs (11): Lastly, to assess the fundamental themes of well-being and overall health the identified generic domains and their associated rankings were mapped to and understood through Max-Neef's human developmental theory of needs. In addition, missing domains within the developed generic outcome sets were identified.

### Step 1: literature review

The inclusion criteria were as follows: systematic reviews, meta-analysis or reports on outcome sets developed by international consortia containing generic PROMs in adult populations; articles from all countries and geographical locations. We restricted inclusion to PROMs developed by international consortia to ensure that included measures were designed through structured, multi-stakeholder, and cross-national processes. This criterion increased the likelihood of identifying PROMs with high generalizability, cross-cultural applicability, and broader uptake across diverse healthcare settings, in line with the international and harmonization goals of the H2O project. The exclusion criteria were as follow: non-English articles, articles with a primary focus on children or adolescents (aged <19), dissertations, guideline statements, and abstracts only.

#### Search strategy

The review followed the PRISMA protocol. Four databases were used: Medline [PubMed], CINHAL [Ebsco], Cochrane [Cochrane library], and PsycINFO [Ovid]. Examples of preliminary search strategies for Medline [PubMed] include: (“Patient Reported Outcome Measures”[MeSH Terms] OR “patient reported outcome*”[Title/Abstract]) AND “systematic review”[Filter]. (“PROMIS”[Title/Abstract] OR “patient reported outcomes measurement information system”[Title/Abstract]) AND/OR “systematic review”[Filter], “International Consortium for Health Outcomes Measurement” [All Fields].

#### Selection of studies

Two postdoctoral researchers screened the titles and abstracts of all retrieved articles and then screened the full texts of the selected articles according to the inclusion and exclusion criteria. Any discrepancies in the results or disagreements were discussed and resolved until consensus was reached. In addition to database searches, the reference lists of included systematic reviews and core outcome set publications were screened to identify relevant reports or grey literature developed by international consortia. These sources were subjected to the same eligibility criteria and screening process as peer-reviewed articles.

#### Data extraction

The results of this review were then used to establish the background for a Delphi exercise to develop a generic PRO set for the needs of the H2O project. Extraction, filtration, and organization of the reviewed works was completed using Atlas ti® focusing on a pragmatic, patient-focused PRO set for clinical routine. Generic PROs and PROMs were extracted and the frequencies of the disease areas in which they were applied were counted.

Themes covered by the identified PROMs were grouped into 11 key concepts/domains using qualitative thematic analysis (19): mental, physical, social, and overall well-being, general quality of life, pain, sleep, energy/vitality, fatigue, cognition, and self-efficacy. Some PROs, such as cognition and self-efficacy, are rarely incorporated in generic PROMs as primary, standalone domains but rather as subdomains.

### Step 2: Delphi consensus procedure

The Delphi procedure was informed by the preceding literature review (Step 1), which served as a preparatory step to identify relevant PRO domains and PROMs for inclusion in the Delphi survey.

We followed the six stages as published in the protocol (17) and conducted a three-round, multi-stakeholder, (electronic) Delphi consensus study:

1. Establish core team
2. Identify stakeholders and set up the core team
3. Start endorsement process
4. Revisit the existing core outcome sets, outcomes and outcome measures
5. Conduct three-round Delphi exercise (Round 1–3)
6. Hold a consensus meeting and report the core outcome set

A Delphi-consensus study is a systematic, feed-back based method to reach consensus on a certain goal by collecting data from participants using a set of iterative questions, sharing the results of these questions with participants, to then reassess the consecutive questionnaires based on previously collected data. Throughout this process, anonymity is maintained (10).

#### Participant recruitment

Eligible participants were all key stakeholders i.e., patients, healthcare professionals (HCPs), academic researchers, regulators, administrators and policy makers. Participants had to be >18 years old and could not be restricted by a chronic disease type (ex. autoimmune diseases, chronic obstructive pulmonary disease, etc.). Participants were recruited through the H2O network using a convenience sampling method (18).

#### Online Delphi questionnaire development and distribution

The online questionnaire was created using the outcome sets identified in the literature review (see Supplementary file). For Round 2, participants were asked to rate the importance of measuring each PRO using two different formats (1) a single-item question and (2) a comprehensive questionnaire. For each frequency measure, the following questions were asked: “On a scale of 1–10 (“not important at all” to “very important”), how important is it to measure PRO xxxx with a single question?”, “From the scale of 1–10 (“not important at all” to “very important”), how important is it to measure PRO xxx with a comprehensive questionnaire?” The phrasing was designed to assess stakeholder preferences for the mode of measurement, not the importance of the PRO domain itself, which had already been established during earlier phases of the study. Explanations were provided to explain what 'single question' and “comprehensive questionnaire” meant. In addition, lay-language definitions were presented to clarify the construct being rated.

We conducted an online three-round Delphi study (Round 1: *N* = 90, Round 2: *N* = 71, Round 3: *N* = 68) including different stakeholder groups (patients = 33; HCP/researcher = 45; Authority/regulator = 3; Industry = 15) followed by consensus meetings with a smaller group of participants consisting of representatives of each stakeholder groups and H2O project members (*N* = 13).

#### Delphi-rounds and cut-off criteria

The ≥70% threshold for scoring 8 or higher was selected based on established practices in Delphi methodology, reflecting a high level of consensus. To ensure appropriate stakeholder representation, results were weighted across groups: 0.5 for patients, 0.4 for HCPs, 0.3 for public experts, and 0.2 for industry representatives. The stakeholder weights were chosen in consultation with the H2O scientific committee to ensure prioritization of the patient voice while still incorporating perspectives from healthcare professionals, public experts, and industry representatives. Prior to the first round, informed consents, contact information and participant characteristics were collected for administrative purposes such as tracking responses and sending reminders; only the Delphi-coordinator had to know participants' details. All participants were given a two-week period to submit their responses to this first questionnaire, and reminders were sent by email to non-responders.

##### Round 1

PROs were excluded from moving forward to round two surveys when ≥10% of any one stakeholder group chose “Never” for measurement frequency. Moreover, a low threshold of agreement was needed in the first round - frequency measures were included for round two when preferred by more than 13% of the patient/patient representative, more than 15% of the clinician/researcher, and more than 15% of the authority AND industry. New frequency measures were included in round 2 when suggested by more than two participants in the free text comment fields, Comments fields were available throughout the questionnaire to allow open-ended feedback. These comments were reviewed qualitatively to identify areas of ambiguity or emerging themes.

##### Round 2& 3

In the second and third rounds, an anonymous report of the results of the previous round were presented to all responders, consisting of the distribution of scores (%) for each. All results were shown separately for patients (including informal caregivers), industry, and public stakeholders (academic researchers/clinicians, and regulators. Then, participants were asked to reassess elements which did not reach consensus in the preceding round. Participants were asked how important it is to measure a specific PRO with a specific frequency measure for a short and/or comprehensive questionnaire, respectively. Participants ranked the importance of different frequencies on a scale of 1–10 (“not important at all” to “very important”). The results were consolidated using a consensus threshold of ≥70% scoring 8 or higher. Feasibility was assessed using the same scaling. The patient stakeholder group results were prioritized for outcome domains and timings by assigning weights (0.5 patients, 0.4 HCPs, 0.3 public experts, and 0.2 industry).

After the three rounds of prioritization, the highest ranked frequency per stakeholder was chosen to be included in the final list to be discussed in the consensus meeting, allowing for final reasons and arguments for potential adaptations.

#### Ethical considerations

Ethical approval was obtained (EK 1803/2021).

### Step 3: H2O mapping exercise

Generic outcome domains identified in the Delphi consensus procedure were cross-mapped to the developed disease-specific H2O PRO outcome sets for lung (13) and metastatic breast cancer (14), diabetes (12) and inflammatory bowel diseases (15). Ranked in order of most shared/overlapping to least, the domains were then compared to two existing and well-established generic outcome frameworks: PROMIS (generic outcomes across diseases) and the EORTC (generic outcome across different cancer entities).

### Step 4: human needs exploration following Max Neef's theory of needs

This mapping exercise used the selected domains from the generic Delphi exercise to explore areas of redundancy and thus assumed importance in the field, as well as gaps, and thus inferred unimportance (11). In doing so, the aim was to highlight the fundamental attributes currently considered key to human well-being as well as to identify which are not represented in the sets reviewed. The Max-Neef model of human needs was selected for this task as it is the only well-validated model which envisions human needs as intersectional and interactive, rather than hierarchical (20). This was considered to be a very strong attribute such that it reflects the real-world more accurately. The model organizes fundamental human needs such as subsistence, protection, affection, understanding, participation, recreation, creation, identity, and freedom. These comprise the *Y*-axis. Then, these needs are also defined according to the existential categories of being, having, doing, and interacting (*X*-axis). Therefore, the model has two axes with specific characteristics falling at the intersection. For example, the intersection of *Protection* and *Having is* rights & social security.

Two researchers AR & PL, psychologists experienced in outcomes research, separately mapped the outcomes to the model and discussed possible implications. Each researcher conducted the mapping independently first without awareness of the other's decisions. A few misalignments occurred and conflict areas triggered a discussion which led to the resolution of conflicts and a final consensus.

## Results

**Figure 1:**
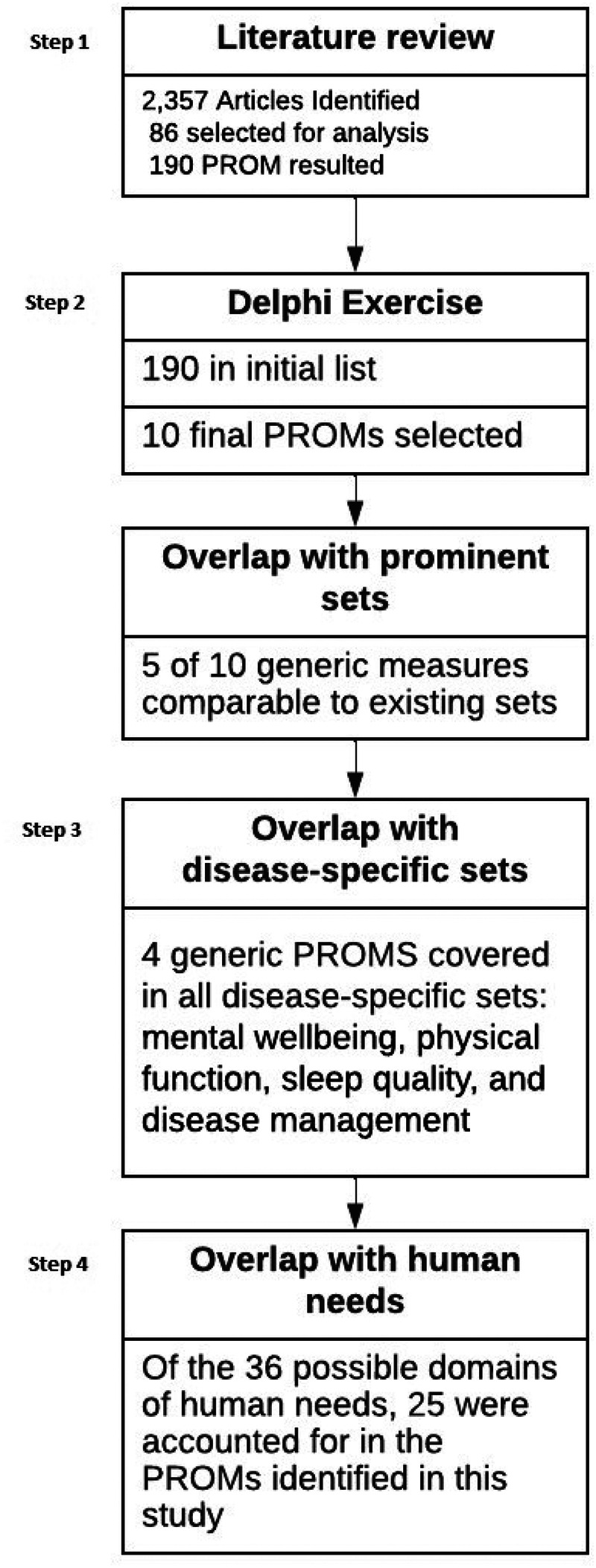
Flow of the methodology.

Results of all four steps are described below, and an overview of the flow of results is presented in Figure 1.

### Step 1: literature review

2,357 articles were identified of which 86 were considered for analysis (see Figure 1). For a complete list of the returned articles please see Supplementary Appendix Table S4. Our analysis showed that around 190 PROMs were considered generic and were administered in all disease areas listed in the International Classification of Diseases, Tenth Revision (ICD-10). Even though well-established cancer-specific PROs exist, in this literature review, cancer is one of the main disease areas in which generic PROs were applied.

Of the 190 PROMs that were found, only 10 had wide domain (more than 5 constructs) and application (more than 3 disease areas) coverage. The three most frequently applied areas of generic PROMs: Disease of the circulatory system, Neoplasms (cancer), and Surgery. The terminologies used for describing domains did not seem to be unified and variations were common within their definitions. In the end, ten generic PROs were identified, however, terminologies differ between established sets (ex. fatigue, energy, and vitality).

**Figure 2:**
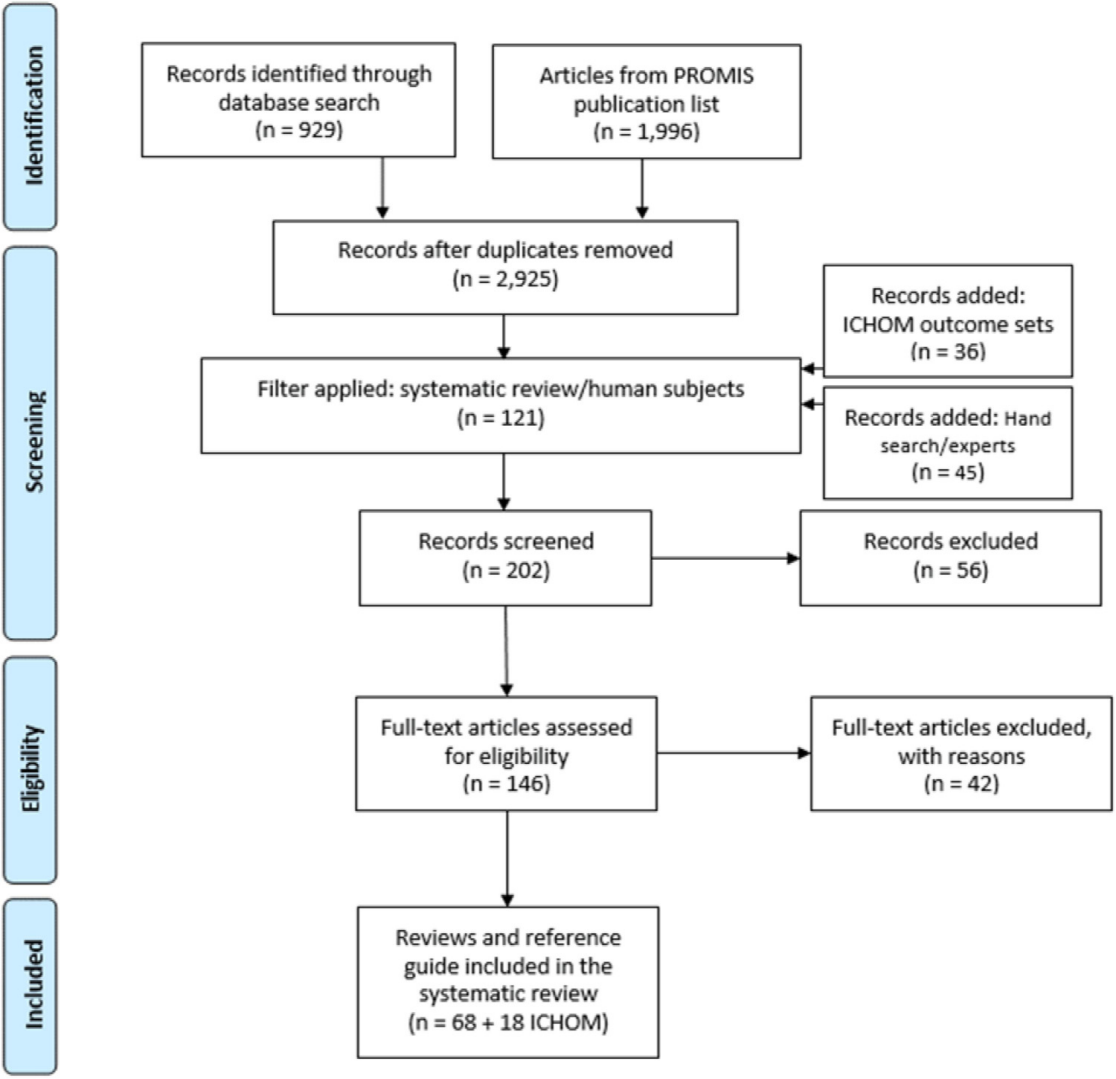
Flow of the Scoping Literature Review.

Of these ten, five generic PROMs were identified with wide domain coverage: PROMIS-10, WHOQoL-BREF, SF-12/36, NHP, EORTC QLQ-C30. Overall, generic outcome sets are applied in a broad range of diseases (*n* = 86) with cancer being the most frequently reported. For a complete overview of the systematic literature review please see Figure 2.

### Step 2: Delphi consensus procedure

Participants in the Delphi study included healthcare professionals (HCP), regulators, patients, administrators, and authorities from four European countries (Austria, Germany, the Netherlands, Spain). The consensus process as reported in the methods section took ten months to complete (January – October 2022). The Delphi exercise resulted in ten final core outcomes. A list of the domains and a brief description can be seen in Supplementary Appendix Table S1. Assessment frequency was consented as every six months per domain. It is important to note that sexuality, self-efficacy, and treatment satisfaction were separately added to the list due to Delphi participants' demand (especially HCPs and patients) although not identified during the literature search.

### Step 3: H2O mapping exercise

Results of the mapping process show that a large number of the generic outcome domains cover specific diseases. Of the core 10 domains identified, four were addressed in every included disease: mental well-being, physical function, sleep quality, and disease management. The overall least reflected domain per unique disease was 'social wellbeing'. This core outcome of the generic set was only included in lung and metastatic breast cancer. The frequency of collection also varied, with disease-specific sets recommended to be assessed at a higher interval (e.g., before each cancer treatment cycle; see Supplementary Appendix Table S2).

A comparison of the H2O generic outcome set and the generic outcome frameworks of the PROMIS and the EORTC can be seen in Supplementary Appendix Table S3. The pragmatic H2O generic outcome set included all core domains of the EORTC and PROMIS and some of the PROMIS sub-domains. Only treatment satisfaction was not seen as individual categories within PROMIS or the EORTC.

### Step 4: human needs exploration following Max Neef's theory of needs

The comparison of Max-Neef's needs model to the output from the Delphi study (see Figure 1) highlights the areas well represented and the areas potentially disregarded at present in the scientific community of generic health outcomes research. The majority of the generic outcomes were assigned to the quality of being rather than associated settings, items, and actions. To illustrate, the quality or experience of health was accounted for by multiple outcomes but none addressed the availability of food (items) or opportunities for rest (setting), both of which could be a more direct predictor. More abstract needs are least represented, such as freedom, identity, and leisure. An emphasis on the qualities or attributes associated with the domains is consistently inquired, but the environmental and material factors are not (see Supplementary Appendix Table S4). Lastly, higher-order needs such as freedom and identity were not represented and thus were either assumed to be of lesser importance or were possibly not considered at all.

## Discussion

This study found that while generic sets offer unique value distinct from disease-specific sets, there are gaps in the currently utilized generic sets which limit their ability to provide a truly complete, general profile of a patient and their health. This study showed that many generic outcome frameworks and measures share the same underlying theoretical construct. Even though differences were detectable, most of these seemed to be due to different semantic definitions or unique disease contexts (e.g., EORTC QLQ-C30) (9). Our findings are similar to other previously developed generic outcome sets (21, 22) but add further domains that seemed especially important and relevant to patients, such as treatment satisfaction.

Including generic outcomes sets into PRO assessment and implementation strategies might especially facilitate quality assessment in clinical settings as well as comparison of disease areas and the establishment of health data spaces (23); such as the H2O project (1) aims to develop.

On a methodological level, a common metrics models could potentially be developed on the base of this project's generic PRO framework (24). Common metric models allow for a centralized conversion of different scales and metrics under one common metric (25). In doing so, different PROMs could be used to assess generic PROs per sites or across sites, e.g., due to the PROMs relevance or establishment in the field, and still be comparable within shared analyses. Focusing on the PRO domain, rather than the instrument (i.e., PROM) will support data comparability and sharing for healthcare in the future.

### Strengths & limitations

A strength of this study was mixed-methods approach to develop the generic PRO set and to consequently test the findings by mapping them to existing generic as well as disease-specific outcomes sets. We also included multiple stakeholders from different fields and disease areas in the procedure and weighted stakeholder results in the Delphi procedure, prioritizing the patients' results to ensure a strong representation of the patient voice. The findings of this study are limited due to the small number of disease areas included. The selected chronic diseases reflect the initial use-cases of interest to the H2O project. Furthermore, the generic analysis focused on two of the most predominant measure frameworks (EORTC and PROMIS) in use, but should be expanded to include lesser-known scales as well. It would have also been beneficial to include the human needs model prior to initiation of the Delphi study to provide a more holistic framework from the start, rather than simply as an evaluative method upon completion. Finally, the inclusion of more patient stakeholders, from more countries and cultures, would help ensure the generalizability of our findings. While we consider the multiple countries a strength of this work, they certainly do not represent an exhaustive list of cultures and backgrounds. The developed outcome set should be further validated in more diverse, particularly non-European contexts. This project focused on the development of a generic PRO outcomes set that assesses general health across diseases. In many contexts, however, it is advisable to complement this generic health set with disease or treatment specific PROs, to ensure a comprehensive representation of the patient's health status.

### Future directions

Life expectancy and the prevalence of chronic diseases and multimorbidity (26) has (with some small exceptions) been on the rise for the last 200 years, leading to significant demographic shifts, and overall changes in treatment targets (27, 28). The aim is now not only to prolong life but to focus on improving patients' quality of life and their subjectively experienced health status while living with a chronic disease. This implies (1) that the recording of the patient's perspective will play an increasingly important role in medical assessments in the future and (2) that data health spaces must be established to store and disseminate the information available. Collecting patient-reported data at scale and making them available together with other data for healthcare and further purposes is at the heart of the H2O project. To further facilitate PRO assessment and to reduce patient and administrative burden, digital solutions and modern psychometric methods like computer-adaptive testing (CAT) are valuable tools.

Within the H2O project, we have successfully created a sound, generic core outcome set for use across different diseases and settings. A key takeaway from this research is that while it is clear that generic sets are highly pragmatic, they do not fully cover any single disease domain across all relevant outcomes, and should be implemented together with a selection of disease-specific outcomes (e.g., hypoglycaemia in diabetes). Similarly, while not a part of either generic or disease-specific PROs, sociodemographic and context factors are relevant to consider when interpreting PROs. The adoption of this set in clinical practice will come with pros and cons. Primarily, any new outcome set requires resources for implementation and requires continued use before longitudinal findings will be possible. However, in contrast, HCPs will gain a more comprehensive profile of their patients that considers their overall wellbeing and can be used for comparison across disease areas.

## Conclusions

Using a four-step mixed-methods approach including a literature review, a three round, multi-stakeholder Delphi consensus procedure in an international diverse group of stakeholders, and several mapping exercises, we developed and analyzed a generic PRO framework aiming to measure health-related quality of life and functioning across diseases. This work resulted in a novel, The final outcome set included in 10 domains (overall health status, mental wellbeing, physical wellbeing, social wellbeing, fatigue, pain, sleep quality, sexuality, self-efficacy, treatment satisfaction) relevant for different disease areas, stakeholder groups and settings. Lastly, the literature review and human needs model comparison has highlighted a gap in outcome collection, specifically on tracking factors that may seem difficult for an HCP to influence, such as resource availability.

## Data Availability

The raw data supporting the conclusions of this article will be made available by the authors, without undue reservation.
