# Supplementary material for: Standardizing patient-reported outcomes across diseases: development of a novel generic patient-reported outcome set
Source: Front Health Serv. 2025 Oct 2;5:1497055. doi: 10.3389/frhs.2025.1497055 (PMC12528166; doi:10.3389/frhs.2025.1497055)
Supplement: Supplementary file 1 [file Table1.docx]

Appendix 1

| **PRO Domain** | | **Construct in lay language** |
| --- | --- | --- |
| 1 | Overall health status | A person’s general physical, mental, and social wellbeing. It reflects the presence or absence of dysfunctions, symptoms or impairment and is also one’s health-related quality of life^1.^ |
| 2 | Mental wellbeing | A person’s psychological health status including among others emotions, mood, stress, fear, anxiety, depression, coping, sense of coherence, and body images – both positive and negative. |
| 3 | Physical wellbeing | A person’s overall physical health mostly related to physical functioning i.e., ability to perform both basic and instrumental activities of daily living without or with minimum support. |
| 4 | Social wellbeing | A person‘s level of participation in social life, such as taking part in family events, other social activities or fulfilling work responsibilities – in general but also specifically related to limitations due to chronic disease^2^. |
| 5 | Fatigue | A sense of persistent tiredness or exhaustion that is often distressing to the individual. It is closely linked to the energy level. In the absence of fatigue, a person might feel totally active or alive. |
| 6 | Pain | A person’s subjective experience of physical pain including specifically its degree and/or severity. |
| 7 | Sleep quality | A person’s perceived personal control over their sleep quality including depth and restoration. |
| 8 | Sexuality | A person’s overall sexuality including different aspects, such as satisfaction with their sex life, physical and emotional sexual functioning, as well as sexual identity and orientation. |
| 9 | Self-efficacy | A person‘ perceived personal control over their health in general as well as their perceived capability for managing (general or health-related) issues. |
| 10 | Treatment satisfaction | A person’s subjective assessment of how satisfied they are with their treatment regimen in general (including medication). This is closely related to the perceived burden of a treatment (e.g. side effects) and the reaching of treatment goals. |

**1** A construct purely describing overall quality of life is not included in the list as it was too general tob e included and operationalized in the H2O observatory. Ex. Satisfaction with Life Scale (example statement: I am statisfied with my life);

**2** Social wellbeing does not include ‚social support‘ as this construct is covered in domains of ‚physical‘ (for tangible and instrumental support) and ‚mental‘ (for emotional support) wellbeing.
