# Supplementary material for: Standardizing patient-reported outcomes across diseases: development of a novel generic patient-reported outcome set
Source: Front Health Serv. 2025 Oct 2;5:1497055. doi: 10.3389/frhs.2025.1497055 (PMC12528166; doi:10.3389/frhs.2025.1497055)
Supplement: Supplementary file 2 [file Table2.docx]

**Appendix 2:** H2O disease comparisons by generic outcome set

| **Generic Outcome Set** | **Diabetes** | **Metastatic breast cancer** | **Lung Cancer** | **IBD** | **Coverage** |
| --- | --- | --- | --- | --- | --- |
| **Overall wellbeing/Quality of Life (QoL)** |  | x |  |  | 75% |
| Health status | x | x | x |  |  |
| Health-related QoL | x | x | x |  |  |
| General QoL |  | x | x |  |  |
| **Mental wellbeing** | x |  |  |  | 100% |
| Emotional functioning |  | x | x | x |  |
| Depression | x | x | x |  |  |
| Stress and anxiety |  | x | x | x |  |
| Cognitive functioning |  | x | x |  |  |
| Body image |  |  |  | x |  |
| **Physical function** |  | x | x | x | 100% |
| Activities of daily living |  | x | x |  |  |
| Mobility |  | x | x |  |  |
| (Diabetes-specific restrictions) | x |  |  |  |  |
| **Social wellbeing** |  |  |  |  | 25% |
| Daily functioning |  | x | x |  |  |
| Social functioning |  | x | x |  |  |
| Role functioning |  | x | x |  |  |
| Relationship (Marital problems) |  | x | x |  |  |
| **Fatigue** |  | x | x | x | 75% |
| **Pain** |  | x | x | x | 75% |
| **Sleep quality** | x |  |  | x | 100% |
| Insomnia |  | x | x |  |  |
| **Sexual function** | x | x |  | x | 75% |
| **Disease management** | x | x | x | x | 100% |
| **Treatment satisfaction** | x |  |  | x | 50% |
| **Work productivity** |  | x |  | x | 50% |
| **Other symptoms** | x | x | x | x | 100% |

*IBD: inflammatory bowel disease; QoL: Quality of life*
