# Supplementary material for: Standardizing patient-reported outcomes across diseases: development of a novel generic patient-reported outcome set
Source: Front Health Serv. 2025 Oct 2;5:1497055. doi: 10.3389/frhs.2025.1497055 (PMC12528166; doi:10.3389/frhs.2025.1497055)
Supplement: Supplementary file 3 [file Table3.docx]

**Appendix 3**: Comparing H2O generic outcome set with other generic PRO frameworks

| **Domains** | **H2O generic outcome set developed in the Delphi exercise** | **PROMIS domains** | **EORTC domains according to the EORTC QLQ-C30** |
| --- | --- | --- | --- |
|  | Overall health status |  | Global Health Status |
|  | Mental wellbeing |  | Emotional Functioning |
|  |  | Anger* |  |
|  |  | Anxiety |  |
|  |  | Depression |  |
|  | Physical functioning | Physical Function | Physical Functioning |
|  | Social wellbeing | Satisfaction with Participation in Social Roles | Social Functioning |
|  |  | Satisfaction with Social Roles & Activities* |  |
|  |  | Ability to Participate in Social Roles & Activities* | Role Functioning |
|  |  | Social Support* |  |
|  |  | Social Isolation* |  |
|  |  | Companionship* |  |
|  | Fatigue | Fatigue | Fatigue |
|  | Pain | Pain Intensity | Pain |
|  |  | Pain Interference |  |
|  |  | Pain Behavior* |  |
|  | Sleep quality | Sleep disturbance |  |
|  |  | Sleep-related impairment* |  |
|  |  |  | Insomnia |
|  | Sexuality | Sexual Function* |  |
|  | Self-efficacy |  |  |
|  |  |  | Nausea/Vomiting |
|  |  |  | Dyspnoea |
|  | Treatment satisfaction |  |  |
|  |  | Applied Cognition* | Cognitive Functioning |
|  |  | Alcohol Use, Consequences, & Expectancies* |  |
|  |  |  | Diarrhea |
|  |  |  | Constipation |
|  |  |  | Appetite loss |
|  |  | Psychosocial Illness Impact* |  |
|  |  |  | Financial Problems |

*PROMIS sub-domains
