# Supplementary material for: Standardizing patient-reported outcomes across diseases: development of a novel generic patient-reported outcome set
Source: Front Health Serv. 2025 Oct 2;5:1497055. doi: 10.3389/frhs.2025.1497055 (PMC12528166; doi:10.3389/frhs.2025.1497055)
Supplement: Supplementary file 4 [file Table4.docx]

Appendix 4: Mapping PRO domains to Max-Neef’s model of human needs

|  | **Needs** | **Qualities** | **Things** | **Actions** | **Settings** |
| --- | --- | --- | --- | --- | --- |
| 1 | **subsistence** | Overall health status  Mental wellbeing  Physical wellbeing  Fatigue  Pain  Sleep quality | **x** | **x** | **x** |
| 2 | **protection** | Overall health status  Physical wellbeing  Self-efficacy  Treatment satisfaction | Social wellbeing | Social wellbeing  Self-efficacy | Social wellbeing |
| 3 | **affection** | Overall health status  Sexuality | Social wellbeing  Sexuality | Social wellbeing  Sexuality | Social wellbeing  Sexuality |
| 4 | **understanding** | Overall health status  Treatment satisfaction | Treatment satisfaction | Treatment satisfaction | **x** |
| 5 | **participation** | Overall health status | Social wellbeing  Self-efficacy | Social wellbeing  Self-efficacy | Social wellbeing |
| 6 | **leisure** | Overall health status | **x** | **x** | **x** |
| 7 | **creation** | Overall health status | Self-efficacy | Self-efficacy | Treatment satisfaction |
| 8 | **identity** | Overall health status  Mental wellbeing  Sexuality | **x** | Sexuality | **x** |
| 9 | **Freedom** | Overall health status  Self-efficacy | **x** | Self-efficacy | **x** |
