# Supplementary material for: Standardizing patient-reported outcomes across diseases: development of a novel generic patient-reported outcome set
Source: Front Health Serv. 2025 Oct 2;5:1497055. doi: 10.3389/frhs.2025.1497055 (PMC12528166; doi:10.3389/frhs.2025.1497055)
Supplement: Supplementary file 5 [file Datasheet5.docx]

**Appendix 5:** Results of the literature review

Abma, I. L., van der Wees, P. J., Veer, V., Westert, G. P., & Rovers, M. (2016). Measurement properties of patient-reported outcome measures (PROMs) in adults with obstructive sleep apnea (OSA): A systematic review. *Sleep Medicine Reviews*, *28*, 18-31. https://doi.org/10.1016/j.smrv.2015.07.006

Ahmed-Lecheheb, D., & Joly, F. (2016). Ovarian cancer survivors’ quality of life: a systematic review. *Journal of Cancer Survivorship*, *10*(5), 789-801. https://doi.org/10.1007/s11764-016-0525-8

Akinosoglou, K., Antonopoulou, S., Katsarolis, I., & Gogos, C. A. (2020). Patient-reported outcomes in HIV clinical trials evaluating antiretroviral treatment: a systematic review. *AIDS Care*, *33*(9), 1118-1126. https://doi.org/10.1080/09540121.2020.1852160

Alsubheen, S. A., Wshah, A., Goldstein, R., & Brooks, D. (2021). Psychometric Properties of Patient-Reported Outcome Measures Assessing Self-Efficacy in Patients with Chronic Obstructive Pulmonary Disease (COPD): A Systematic Review. *COPD: Journal of Chronic Obstructive Pulmonary Disease*, *18*(2), 254-263. https://doi.org/10.1080/15412555.2021.1897559

Atkinson, T. M., Andreotti, C. F., Roberts, K. E., Saracino, R. M., Hernandez, M., & Basch, E. (2015). The level of association between functional performance status measures and patient-reported outcomes in cancer patients: a systematic review. *Supportive Care in Cancer*, *23*(12), 3645-3652. https://doi.org/10.1007/s00520-015-2923-2

Ayton, D. R., Gardam, M. L., Pritchard, E. K., Ruseckaite, R., Ryan, J., Robinson, S. J., Brodaty, H., Ward, S. A., Ahern, S., & Heyn, P. C. (2021). Patient-Reported Outcome Measures to Inform Care of People With Dementia—A Systematic Scoping Review. *The Gerontologist*, *61*(5), e185-e194. https://doi.org/10.1093/geront/gnz179

Beatty, L., Kemp, E., Butow, P., Girgis, A., Schofield, P., Turner, J., Hulbert‐Williams, N. J., Levesque, J. V., & Koczwara, B. (2017). A systematic review of psychotherapeutic interventions for women with metastatic breast cancer: Context matters. *Psycho-Oncology*, *27*(1), 34-42. https://doi.org/10.1002/pon.4445

Buchanan, A., Roddy, M., & Badr, H. (2020). Patient-reported outcomes of non-pharmacological interventions for endometrial cancer survivors: a systematic review. *Journal of Cancer Survivorship*, *15*(4), 526-535. https://doi.org/10.1007/s11764-020-00946-z

Bunevicius, A., Miller, J., & Parsons, M. (2020). Isocitrate Dehydrogenase, Patient-Reported Outcomes, and Cognitive Functioning of Glioma Patients: a Systematic Review. *Current Oncology Reports*, *22*(12). https://doi.org/10.1007/s11912-020-00978-9

Burns, D. J. P., Arora, J., Okunade, O., Beltrame, J. F., Bernardez-Pereira, S., Crespo-Leiro, M. G., Filippatos, G. S., Hardman, S., Hoes, A. W., Hutchison, S., Jessup, M., Kinsella, T., Knapton, M., Lam, C. S. P., Masoudi, F. A., McIntyre, H., Mindham, R., Morgan, L., Otterspoor, L., Parker, V., Persson, H. E., Pinnock, C., Reid, C. M., Riley, J., Stevenson, L. W., & McDonagh, T. A. (2020). International Consortium for Health Outcomes Measurement (ICHOM): Standardized Patient-Centered Outcomes Measurement Set for Heart Failure Patients. *JACC: Heart Failure*, *8*(3), 212-222. https://doi.org/10.1016/j.jchf.2019.09.007

Cañete, J. D., Tasende, J. A. P., Laserna, F. J. R., Castro, S. G., & Queiro, R. (2020). The Impact of Comorbidity on Patient-Reported Outcomes in Psoriatic Arthritis: A Systematic Literature Review. *Rheumatology and Therapy*, *7*(2), 237-257. https://doi.org/10.1007/s40744-020-00202-x

Carlozzi, N. E., Miciura, A., Migliore, N., & Dayalu, P. (2014). Understanding the Outcomes Measures used in Huntington Disease Pharmacological Trials: A Systematic Review. *Journal of Huntington's Disease*, *3*(3), 233-252. https://doi.org/10.3233/jhd-140115

Chan, A., Cameron, M. C., Garden, B., Boers-Doets, C. B., Schindler, K., Epstein, J. B., Choi, J., Beamer, L., Roeland, E., Russi, E. G., Bensadoun, R.-J., Teo, Y. L., Chan, R. J., Shih, V., Bryce, J., Raber-Durlacher, J., Gerber, P. A., Freytes, C. O., Rapoport, B., LeBoeuf, N., Sibaud, V., & Lacouture, M. E. (2015). A systematic review of patient-reported outcome instruments of dermatologic adverse events associated with targeted cancer therapies. *Supportive Care in Cancer*, *23*(8), 2231-2244. https://doi.org/10.1007/s00520-014-2564-x

Chen, R. C., Chang, P., Vetter, R. J., Lukka, H., Stokes, W. A., Sanda, M. G., Watkins-Bruner, D., Reeve, B. B., & Sandler, H. M. (2014). Recommended Patient-Reported Core Set of Symptoms to Measure in Prostate Cancer Treatment Trials. *JNCI: Journal of the National Cancer Institute*, *106*(7). https://doi.org/10.1093/jnci/dju132

Chen, Y. T., Tan, Y. Z., Cheen, M., & Wee, H.-L. (2019). Patient-Reported Outcome Measures in Registry-Based Studies of Type 2 Diabetes Mellitus: a Systematic Review. *Current Diabetes Reports*, *19*(11). https://doi.org/10.1007/s11892-019-1265-8

Cherkaoui, Z., González, C., Wakabayashi, T., Delattre, B., Léost, E., Serra, S., ... & Pessaux, P. (2021). A standard set of value-based patient-centered outcomes for pancreatic carcinoma: an international delphi survey. *Annals of Surgical Oncology*, *28*, 1069-1078. Colomer-Lahiguera, S., Bryant-Lukosius, D., Rietkoetter, S., Martelli, L., Ribi, K., Fitzpatrick-Lewis, D., Sherifali, D., Orcurto, A., Juergens, R., & Eicher, M. (2020). Patient-reported outcome instruments used in immune-checkpoint inhibitor clinical trials in oncology: a systematic review. *Journal of Patient-Reported Outcomes*, *4*(1). https://doi.org/10.1186/s41687-020-00210-z

de Jong, M. J., Huibregtse, R., Masclee, A. A. M., Jonkers, D. M. A. E., & Pierik, M. J. (2018). Patient-Reported Outcome Measures for Use in Clinical Trials and Clinical Practice in Inflammatory Bowel Diseases: A Systematic Review. *Clinical Gastroenterology and Hepatology*, *16*(5), 648-663.e643. https://doi.org/10.1016/j.cgh.2017.10.019

de Roos, P., Bloem, B. R., Kelley, T. A., Antonini, A., Dodel, R., Hagell, P., Marras, C., Martinez-Martin, P., Mehta, S. H., Odin, P., Chaudhuri, K. R., Weintraub, D., Wilson, B., & Uitti, R. J. (2017). A Consensus Set of Outcomes for Parkinson’s Disease from the International Consortium for Health Outcomes Measurement. *Journal of Parkinson's Disease*, *7*(3), 533-543. https://doi.org/10.3233/jpd-161055

Djan, R., & Penington, A. (2013). A systematic review of questionnaires to measure the impact of appearance on quality of life for head and neck cancer patients. *Journal of Plastic, Reconstructive & Aesthetic Surgery*, *66*(5), 647-659. https://doi.org/10.1016/j.bjps.2013.01.007

Doganay Erdogan, B., Leung, Y. Y., Pohl, C., Tennant, A., & Conaghan, P. G. (2016). Minimal Clinically Important Difference as Applied in Rheumatology: An OMERACT Rasch Working Group Systematic Review and Critique. *The Journal of Rheumatology*, *43*(1), 194-202. https://doi.org/10.3899/jrheum.141150

Domb, B. G., Gui, C., & Lodhia, P. (2015). How Much Arthritis Is Too Much for Hip Arthroscopy: A Systematic Review. *Arthroscopy: The Journal of Arthroscopic & Related Surgery*, *31*(3), 520-529. https://doi.org/10.1016/j.arthro.2014.11.008

Ekström, M., Sundh, J., & Larsson, K. (2016). Patient reported outcome measures in chronic obstructive pulmonary disease: Which to use? *Expert Review of Respiratory Medicine*, *10*(3), 351-362. https://doi.org/10.1586/17476348.2016.1146595

Engler, K., Lessard, D., & Lebouché, B. (2016). A Review of HIV-Specific Patient-Reported Outcome Measures. *The Patient - Patient-Centered Outcomes Research*, *10*(2), 187-202. https://doi.org/10.1007/s40271-016-0195-7

Faithfull, S., Turner, L., Poole, K., Joy, M., Manders, R., Weprin, J., Winters‐Stone, K., & Saxton, J. (2019). Prehabilitation for adults diagnosed with cancer: A systematic review of long‐term physical function, nutrition and patient‐reported outcomes. *European Journal of Cancer Care*, *28*(4). https://doi.org/10.1111/ecc.13023

Foust-Wright, C., Wissig, S., Stowell, C., Olson, E., Anderson, A., Anger, J., Cardozo, L., Cotterill, N., Gormley, E. A., Toozs-Hobson, P., Heesakkers, J., Herbison, P., Moore, K., McKinney, J., Morse, A., Pulliam, S., Szonyi, G., Wagg, A., & Milsom, I. (2017). Development of a core set of outcome measures for OAB treatment. *International Urogynecology Journal*, *28*(12), 1785-1793. https://doi.org/10.1007/s00192-017-3481-6

Galantino, M. L., Eden, M. M., Spinelli, B. A., & Flores, A. M. (2015). EDGE Task Force on Head and Neck Cancer Outcomes: a systematic review of outcome measures for temporomandibular-related dysfunction. *Rehabilitation Oncology*, *33*(2), 6-14.

Giesinger, J. M., Efficace, F., Aaronson, N., Calvert, M., Kyte, D., Cottone, F., Cella, D., & Gamper, E.-M. (2021). Past and Current Practice of Patient-Reported Outcome Measurement in Randomized Cancer Clinical Trials: A Systematic Review. *Value in Health*, *24*(4), 585-591. https://doi.org/10.1016/j.jval.2020.11.004

Go, V. A. A., Thomas, M. C., Singh, B., Prenatt, S., Sims, H., Blanck, J. F., & Segars, J. H. (2020). A systematic review of the psychosocial impact of fibroids before and after treatment. *American Journal of Obstetrics and Gynecology*, *223*(5), 674-708.e678. https://doi.org/10.1016/j.ajog.2020.05.044

Harrington, S., Lee, J., Colon, G., & Alappattu, M. (2016). Oncology Section EDGE Task Force on Prostate Cancer. *Rehabilitation Oncology*, *34*(1), 27-35. https://doi.org/10.1097/01.Reo.0000000000000004

Hayes, B., Moller, S., Wilding, H., Burgell, R., Apputhurai, P., & Knowles, S. R. (2020). Application of the common sense model in inflammatory bowel disease: A systematic review. *Journal of Psychosomatic Research*, *139*. https://doi.org/10.1016/j.jpsychores.2020.110283

Haywood, K. L., Pearson, N., Morrison, L. J., Castrén, M., Lilja, G., & Perkins, G. D. (2018). Assessing health-related quality of life (HRQoL) in survivors of out-of-hospital cardiac arrest: A systematic review of patient-reported outcome measures. *Resuscitation*, *123*, 22-37. https://doi.org/10.1016/j.resuscitation.2017.11.065

Hendrikx, J., de Jonge, M. J., Fransen, J., Kievit, W., & van Riel, P. L. C. M. (2016). Systematic review of patient-reported outcome measures (PROMs) for assessing disease activity in rheumatoid arthritis. *RMD Open*, *2*(2). https://doi.org/10.1136/rmdopen-2015-000202

Hepworth, L. R., Rowe, F. J., Harper, R., Jarvis, K., Shipman, T., & Rodgers, H. (2015). Patient reported outcome measures for visual impairment after stroke: a systematic review. *Health and Quality of Life Outcomes*, *13*(1). https://doi.org/10.1186/s12955-015-0338-x

Hess, L. M., & Stehman, F. B. (2012). State of the Science in Ovarian Cancer Quality of Life Research. *International Journal of Gynecological Cancer*, *22*(7), 1273-1280. https://doi.org/10.1097/IGC.0b013e318263f02e

Hogg, F. R. A., Peach, G., Price, P., Thompson, M. M., & Hinchliffe, R. J. (2012). Measures of health-related quality of life in diabetes-related foot disease: a systematic review. *Diabetologia*, *55*(3), 552-565. https://doi.org/10.1007/s00125-011-2372-5

Houts, C. R., McGinley, J. S., Nishida, T. K., Buse, D. C., Wirth, R. J., Dodick, D. W., Goadsby, P. J., & Lipton, R. B. (2021). Systematic review of outcomes and endpoints in acute migraine clinical trials. *Headache: The Journal of Head and Face Pain*, *61*(2), 263-275. https://doi.org/10.1111/head.14067

Hurd, K., & Barnabe, C. (2016). Systematic review of rheumatic disease phenotypes and outcomes in the Indigenous populations of Canada, the USA, Australia and New Zealand. *Rheumatology International*, *37*(4), 503-521. https://doi.org/10.1007/s00296-016-3623-z

Hyde, M. K., Chambers, S. K., Shum, D., Ip, D., & Dunn, J. (2015). Psycho‐oncology assessment in Chinese populations: a systematic review of quality of life and psychosocial measures. *European Journal of Cancer Care*, *25*(5), 691-718. https://doi.org/10.1111/ecc.12367

Ijzerman-Korevaar, M., Snijders, T. J., de Graeff, A., Teunissen, S. C. C. M., & de Vos, F. Y. F. (2018). Prevalence of symptoms in glioma patients throughout the disease trajectory: a systematic review. *Journal of Neuro-Oncology*, *140*(3), 485-496. https://doi.org/10.1007/s11060-018-03015-9

Jacobs, M., Macefield, R. C., Blazeby, J. M., Korfage, I. J., van Berge Henegouwen, M. I., de Haes, H. C. J. M., Smets, E. M., & Sprangers, M. A. G. (2012). Systematic review reveals limitations of studies evaluating health-related quality of life after potentially curative treatment for esophageal cancer. *Quality of Life Research*, *22*(7), 1787-1803. https://doi.org/10.1007/s11136-012-0290-8

Kilic, L., Erden, A., Bingham, C. O., Gossec, L., & Kalyoncu, U. (2016). The Reporting of Patient-reported Outcomes in Studies of Patients with Rheumatoid Arthritis: A Systematic Review of 250 Articles. *The Journal of Rheumatology*, *43*(7), 1300-1305. https://doi.org/10.3899/jrheum.151177

Kim, D.-Y., Lee, J.-S., & Son, C.-G. (2020). Systematic Review of Primary Outcome Measurements for Chronic Fatigue Syndrome/Myalgic Encephalomyelitis (CFS/ME) in Randomized Controlled Trials. *Journal of Clinical Medicine*, *9*(11). https://doi.org/10.3390/jcm9113463

Knoble, N., Nayroles, G., Cheng, C., & Arnould, B. (2018). Illustration of patient-reported outcome challenges and solutions in rare diseases: a systematic review in Cushing’s syndrome. *Orphanet Journal of Rare Diseases*, *13*(1). https://doi.org/10.1186/s13023-018-0958-4

Kohn, C. G., Singh, P., Korytowsky, B., Caranfa, J. T., Miller, J. D., Sill, B. E., ... & Parikh, N. D. (2019). Humanistic and economic burden of hepatocellular carcinoma: systematic literature review. *The American Journal of Managed Care*, *25*(2 Spec No.), SP61-SP73.

Korsten, L. H. A., Jansen, F., de Haan, B. J. F., Sent, D., Cuijpers, P., Leemans, C. R., & Verdonck‐de Leeuw, I. M. (2019). Factors associated with depression over time in head and neck cancer patients: A systematic review. *Psycho-Oncology*, *28*(6), 1159-1183. https://doi.org/10.1002/pon.5058

Lee, E. H., Klassen, A. F., Nehal, K. S., Cano, S. J., Waters, J., & Pusic, A. L. (2013). A systematic review of patient-reported outcome instruments of nonmelanoma skin cancer in the dermatologic population. *Journal of the American Academy of Dermatology*, *69*(2), e59-e67. https://doi.org/10.1016/j.jaad.2012.09.017

Lombardi, P., Marandino, L., De Luca, E., Zichi, C., Reale, M. L., Pignataro, D., Di Stefano, R. F., Ghisoni, E., Mariniello, A., Trevisi, E., Leone, G., Muratori, L., La Salvia, A., Sonetto, C., Leone, F., Aglietta, M., Novello, S., Scagliotti, G. V., Perrone, F., & Di Maio, M. (2020). Quality of life assessment and reporting in colorectal cancer: A systematic review of phase III trials published between 2012 and 2018. *Critical Reviews in Oncology/Hematology*, *146*. https://doi.org/10.1016/j.critrevonc.2020.102877

Maguire, R., Kotronoulas, G., Papadopoulou, C., Simpson, M. F., McPhelim, J., & Irvine, L. (2013). Patient-Reported Outcome Measures for the Identification of Supportive Care Needs in People With Lung Cancer. *Cancer Nursing*, *36*(4), E1-E17. https://doi.org/10.1097/NCC.0b013e31826f3c8f

Maguire, R., Papadopoulou, C., Kotronoulas, G., Simpson, M. F., McPhelim, J., & Irvine, L. (2013). A systematic review of supportive care needs of people living with lung cancer. *European Journal of Oncology Nursing*, *17*(4), 449-464. https://doi.org/10.1016/j.ejon.2012.10.013

Manier, K. K., Rowe, L. S., Welsh, J., & Armstrong, T. S. (2018). The impact and incidence of altered body image in patients with head and neck tumors: a systematic review. *Neuro-Oncology Practice*, *5*(4), 204-213. https://doi.org/10.1093/nop/npy018

Marandino, L., De Luca, E., Zichi, C., Lombardi, P., Reale, M. L., Pignataro, D., Di Stefano, R. F., Ghisoni, E., Mariniello, A., Trevisi, E., Leone, G., Muratori, L., La Salvia, A., Sonetto, C., Buttigliero, C., Tucci, M., Aglietta, M., Novello, S., Scagliotti, G. V., Perrone, F., & Di Maio, M. (2019). Quality-of-Life Assessment and Reporting in Prostate Cancer: Systematic Review of Phase 3 Trials Testing Anticancer Drugs Published Between 2012 and 2018. *Clinical Genitourinary Cancer*, *17*(5), 332-347.e332. https://doi.org/10.1016/j.clgc.2019.07.007

Martini, C., Gamper, E.-M., Wintner, L., Nilica, B., Sperner-Unterweger, B., Holzner, B., & Virgolini, I. (2016). Systematic review reveals lack of quality in reporting health-related quality of life in patients with gastroenteropancreatic neuroendocrine tumours. *Health and Quality of Life Outcomes*, *14*(1). https://doi.org/10.1186/s12955-016-0527-2

Matteson, K. A., Boardman, L. A., Munro, M. G., & Clark, M. A. (2009). Abnormal uterine bleeding: a review of patient-based outcome measures. *Fertility and Sterility*, *92*(1), 205-216. https://doi.org/10.1016/j.fertnstert.2008.04.023

McFarland, D. C., Blackler, E., Banerjee, S., & Holland, J. (2017). Communicating about precision oncology. *JCO Precision Oncology*, *1*, 1-9.

McGinley, J. S., Houts, C. R., Nishida, T. K., Buse, D. C., Lipton, R. B., Goadsby, P. J., Dodick, D. W., & Wirth, R. J. (2021). Systematic review of outcomes and endpoints in preventive migraine clinical trials. *Headache: The Journal of Head and Face Pain*, *61*(2), 253-262. https://doi.org/10.1111/head.14069

McLeod, C., Wood, J., Schultz, A., Norman, R., Smith, S., Blyth, C. C., Webb, S., Smyth, A. R., & Snelling, T. L. (2020). Outcomes and endpoints reported in studies of pulmonary exacerbations in people with cystic fibrosis: A systematic review. *Journal of Cystic Fibrosis*, *19*(6), 858-867. https://doi.org/10.1016/j.jcf.2020.08.015

McNamara, R. L., Spatz, E. S., Kelley, T. A., Stowell, C. J., Beltrame, J., Heidenreich, P., Tresserras, R., Jernberg, T., Chua, T., Morgan, L., Panigrahi, B., Rosas Ruiz, A., Rumsfeld, J. S., Sadwin, L., Schoeberl, M., Shahian, D., Weston, C., Yeh, R., & Lewin, J. (2015). Standardized Outcome Measurement for Patients With Coronary Artery Disease: Consensus From the International Consortium for Health Outcomes Measurement (ICHOM). *Journal of the American Heart Association*, *4*(5). https://doi.org/10.1161/jaha.115.001767

Mercieca-Bebber, R. L., Perreca, A., King, M., Macann, A., Whale, K., Soldati, S., Jacobs, M., & Efficace, F. (2016). Patient-reported outcomes in head and neck and thyroid cancer randomised controlled trials: A systematic review of completeness of reporting and impact on interpretation. *European Journal of Cancer*, *56*, 144-161. https://doi.org/10.1016/j.ejca.2015.12.025

Mertz, K., Lindsay, S. E., Morris, A., & Kamal, R. N. (2020). Outcome Metrics in the Treatment of Carpal Tunnel Syndrome: A Systematic Review. *Hand*, *17*(4), 659-667. https://doi.org/10.1177/1558944720949951

Michaud, K., Pope, J., van de Laar, M., Curtis, J. R., Kannowski, C., Mitchell, S., Bell, J., Workman, J., Paik, J., Cardoso, A., & Taylor, P. C. (2021). Systematic Literature Review of Residual Symptoms and an Unmet Need in Patients With Rheumatoid Arthritis. *Arthritis Care & Research*, *73*(11), 1606-1616. https://doi.org/10.1002/acr.24369

Mierzynska, J., Piccinin, C., Pe, M., Martinelli, F., Gotay, C., Coens, C., Mauer, M., Eggermont, A., Groenvold, M., Bjordal, K., Reijneveld, J., Velikova, G., & Bottomley, A. (2019). Prognostic value of patient-reported outcomes from international randomised clinical trials on cancer: a systematic review. *The Lancet Oncology*, *20*(12), e685-e698. https://doi.org/10.1016/s1470-2045(19)30656-4

Minnock, P., McKee, G., Kelly, A., Carter, S. C., Menzies, V., O’Sullivan, D., Richards, P., Ndosi, M., & van Eijk Hustings, Y. (2018). Nursing sensitive outcomes in patients with rheumatoid arthritis: A systematic literature review. *International Journal of Nursing Studies*, *77*, 115-129. https://doi.org/10.1016/j.ijnurstu.2017.09.005

Montazeri, A. (2009). Quality of life data as prognostic indicators of survival in cancer patients: an overview of the literature from 1982 to 2008. *Health and Quality of Life Outcomes*, *7*(1). https://doi.org/10.1186/1477-7525-7-102

Montazeri, A., Gondivkar, S. M., Gadbail, A. R., Sarode, S. C., Gondivkar, R. S., Yuwanati, M., Sarode, G. S., & Patil, S. (2019). Measurement properties of oral health related patient reported outcome measures in patients with oral cancer: A systematic review using COSMIN checklist. *PloS one*, *14*(6). https://doi.org/10.1371/journal.pone.0218833

Monti, S., Quinn, K. A., Christensen, R., Jayne, D., Langford, C., Lanier, G. E., Mahr, A., Pagnoux, C., Viðarsdóttir, M. B., Merkel, P. A., & Tomasson, G. (2020). Use and reporting of outcome measures in randomized trials for anti-neutrophil cytoplasmic antibody-associated vasculitis: a systematic literature review. *Seminars in Arthritis and Rheumatism*, *50*(6), 1314-1325. https://doi.org/10.1016/j.semarthrit.2020.09.010

Morgans, A. K., van Bommel, A. C. M., Stowell, C., Abrahm, J. L., Basch, E., Bekelman, J. E., Berry, D. L., Bossi, A., Davis, I. D., de Reijke, T. M., Denis, L. J., Evans, S. M., Fleshner, N. E., George, D. J., Kiefert, J., Lin, D. W., Matthew, A. G., McDermott, R., Payne, H., Roos, I. A. G., Schrag, D., Steuber, T., Tombal, B., van Basten, J.-P., van der Hoeven, J. J. M., & Penson, D. F. (2015). Development of a Standardized Set of Patient-centered Outcomes for Advanced Prostate Cancer: An International Effort for a Unified Approach. *European Urology*, *68*(5), 891-898. https://doi.org/10.1016/j.eururo.2015.06.007

Nelson, C. J., Cho, C., Berk, A. R., Holland, J., & Roth, A. J. (2010). Are Gold Standard Depression Measures Appropriate for Use in Geriatric Cancer Patients? A Systematic Evaluation of Self-Report Depression Instruments Used With Geriatric, Cancer, and Geriatric Cancer Samples. *Journal of Clinical Oncology*, *28*(2), 348-356. https://doi.org/10.1200/jco.2009.23.0201

Nijagal, M. A., Wissig, S., Stowell, C., Olson, E., Amer-Wahlin, I., Bonsel, G., Brooks, A., Coleman, M., Devi Karalasingam, S., Duffy, J. M. N., Flanagan, T., Gebhardt, S., Greene, M. E., Groenendaal, F., R Jeganathan, J. R., Kowaliw, T., Lamain-de-Ruiter, M., Main, E., Owens, M., Petersen, R., Reiss, I., Sakala, C., Speciale, A. M., Thompson, R., Okunade, O., & Franx, A. (2018). Standardized outcome measures for pregnancy and childbirth, an ICHOM proposal. *BMC Health Services Research*, *18*(1). https://doi.org/10.1186/s12913-018-3732-3

Ogunsanya, M. E., Kalb, S. J., Kabaria, A., & Chen, S. (2016). A systematic review of patient‐reported outcomes in patients with cutaneous lupus erythematosus. *British Journal of Dermatology*, *176*(1), 52-61. https://doi.org/10.1111/bjd.14868

Ortega-Avila, A. B., Ramos-Petersen, L., Cervera-Garvi, P., Nester, C. J., Morales-Asencio, J. M., & Gijon-Nogueron, G. (2019). Systematic review of the psychometric properties of patient-reported outcome measures for rheumatoid arthritis in the foot and ankle. *Clinical Rehabilitation*, *33*(11), 1788-1799. https://doi.org/10.1177/0269215519862328

Palominos, P. E., Gaujoux‐Viala, C., Fautrel, B., Dougados, M., & Gossec, L. (2012). Clinical outcomes in psoriatic arthritis: A systematic literature review. *Arthritis Care & Research*, *64*(3), 397-406. https://doi.org/10.1002/acr.21552

Paschos, P., Katsoula, A., Salanti, G., Giouleme, O., Athanasiadou, E., & Tsapas, A. (2018). Systematic review with network meta‐analysis: the impact of medical interventions for moderate‐to‐severe ulcerative colitis on health‐related quality of life. *Alimentary Pharmacology & Therapeutics*, *48*(11-12), 1174-1185. https://doi.org/10.1111/apt.15005

Poku, E., Aber, A., Phillips, P., Essat, M., Buckley Woods, H., Palfreyman, S., Kaltenthaler, E., Jones, G., & Michaels, J. (2017). Systematic review assessing the measurement properties of patient-reported outcomes for venous leg ulcers. *BJS Open*, *1*(5), 138-147. https://doi.org/10.1002/bjs5.25

Ramsey, I., Eckert, M., Hutchinson, A. D., Marker, J., & Corsini, N. (2020). Core outcome sets in cancer and their approaches to identifying and selecting patient-reported outcome measures: a systematic review. *Journal of Patient-Reported Outcomes*, *4*(1). https://doi.org/10.1186/s41687-020-00244-3

Razvi, S., McMillan, C. V., & Weaver, J. U. (2005). Instruments used in measuring symptoms, health status and quality of life in hypothyroidism: a systematic qualitative review. *Clinical Endocrinology*, *63*(6), 617-624. https://doi.org/10.1111/j.1365-2265.2005.02381.x

Riemer, C. A., el-Azhary, R. A., Wu, K. L., Strand, J. J., & Lehman, J. S. (2017). Underreported use of palliative care and patient-reported outcome measures to address reduced quality of life in patients with calciphylaxis: a systematic review. *British Journal of Dermatology*, *177*(6), 1510-1518. https://doi.org/10.1111/bjd.15702

Rolfson, O., Wissig, S., van Maasakkers, L., Stowell, C., Ackerman, I., Ayers, D., Barber, T., Benzakour, T., Bozic, K., Budhiparama, N., Caillouette, J., Conaghan, P. G., Dahlberg, L., Dunn, J., Grady‐Benson, J., Ibrahim, S. A., Lewis, S., Malchau, H., Manzary, M., March, L., Nassif, N., Nelissen, R., Smith, N., & Franklin, P. D. (2016). Defining an International Standard Set of Outcome Measures for Patients With Hip or Knee Osteoarthritis: Consensus of the International Consortium for Health Outcomes Measurement Hip and Knee Osteoarthritis Working Group. *Arthritis Care & Research*, *68*(11), 1631-1639. https://doi.org/10.1002/acr.22868

Rönsch, H., Apfelbacher, C., Brans, R., Matterne, U., Molin, S., Ofenloch, R., Oosterhaven, J. A. F., Schuttelaar, M. L. A., Weisshaar, E., Yew, Y. W., & Bauer, A. (2019). Which outcomes have been measured in hand eczema trials? A systematic review. *Contact Dermatitis*, *80*(4), 201-207. https://doi.org/10.1111/cod.13212

Rutherford, C., Campbell, R., White, K., & King, M. (2019). Patient-reported outcomes as predictors of survival in patients with bowel cancer: a systematic review. *Quality of Life Research*, *28*(11), 2871-2887. https://doi.org/10.1007/s11136-019-02255-0

Safa, H., Tamil, M., Spiess, P. E., Manley, B., Pow-Sang, J., Gilbert, S. M., Safa, F., Gonzalez, B. D., Oswald, L. B., Semaan, A., Diab, A., & Chahoud, J. (2021). Patient-Reported Outcomes in Clinical Trials Leading to Cancer Immunotherapy Drug Approvals From 2011 to 2018: A Systematic Review. *JNCI: Journal of the National Cancer Institute*, *113*(5), 532-542. https://doi.org/10.1093/jnci/djaa174

Salame, N., Perez-Chada, Lourdes M., Singh, S., Callis Duffin, K., Garg, A., Gottlieb, Alice B., Latella, J., Merola, Joseph F., & Armstrong, April W. (2018). Are Your Patients Satisfied A Systematic Review of Treatment Satisfaction Measures in Psoriasis. *Dermatology*, *234*(5-6), 157-165. https://doi.org/10.1159/000490413

Schouten, B., Avau, B., Bekkering, G. T. E., Vankrunkelsven, P., Mebis, J., Hellings, J., & Van Hecke, A. (2019). Systematic screening and assessment of psychosocial well-being and care needs of people with cancer. *Cochrane Database of Systematic Reviews*. https://doi.org/10.1002/14651858.CD012387.pub2

Seal, B. S., Asche, C. V., Puto, K., & Allen, P. D. (2013). Efficacy, Patient-Reported Outcomes (PROs), and Tolerability of the Changing Therapeutic Landscape in Patients with Metastatic Prostate Cancer (MPC): A Systematic Literature Review. *Value in Health*, *16*(5), 872-890. https://doi.org/10.1016/j.jval.2013.03.1628

Seligman, W. H., Das-Gupta, Z., Jobi-Odeneye, A. O., Arbelo, E., Banerjee, A., Bollmann, A., Caffrey-Armstrong, B., Cehic, D. A., Corbalan, R., Collins, M., Dandamudi, G., Dorairaj, P., Fay, M., Van Gelder, I. C., Goto, S., Granger, C. B., Gyorgy, B., Healey, J. S., Hendriks, J. M., Hills, M. T., Hobbs, F. D. R., Huisman, M. V., Koplan, K. E., Lane, D. A., Lewis, W. R., Lobban, T., Steinberg, B. A., McLeod, C. J., Moseley, S., Timmis, A., Yutao, G., & Camm, A. J. (2020). Development of an international standard set of outcome measures for patients with atrial fibrillation: a report of the International Consortium for Health Outcomes Measurement (ICHOM) atrial fibrillation working group. *European Heart Journal*, *41*(10), 1132-1140. https://doi.org/10.1093/eurheartj/ehz871

Shunmugasundaram, C., Rutherford, C., Butow, P. N., Sundaresan, P., & Dhillon, H. M. (2019). Content comparison of unmet needs self‐report measures used in patients with head and neck cancer: A systematic review. *Psycho-Oncology*, *28*(12), 2295-2306. https://doi.org/10.1002/pon.5257

Shunmugasundaram, C., Rutherford, C., Butow, P. N., Sundaresan, P., & Dhillon, H. M. (2020). What are the optimal measures to identify anxiety and depression in people diagnosed with head and neck cancer (HNC): a systematic review. *Journal of Patient-Reported Outcomes*, *4*(1). https://doi.org/10.1186/s41687-020-00189-7

Skogestad, I. J., Kirkevold, M., Indredavik, B., Gay, C. L., & Lerdal, A. (2019). Lack of content overlap and essential dimensions – A review of measures used for post-stroke fatigue. *Journal of Psychosomatic Research*, *124*. https://doi.org/10.1016/j.jpsychores.2019.109759

Stewart, C. R., Algu, L., Kamran, R., Leveille, C. F., Abid, K., Rae, C., & Lipner, S. R. (2020). Patient Satisfaction with Treatment for Onychocryptosis: A Systematic Review. *Skin Appendage Disorders*, *6*(5), 272-279. https://doi.org/10.1159/000508927

Stewart, C. R., Algu, L., Kamran, R., Leveille, C. F., Abid, K., Rae, C., & Lipner, S. R. (2021). Effect of onychomycosis and treatment on patient-reported quality-of-life outcomes: A systematic review. *Journal of the American Academy of Dermatology*, *85*(5), 1227-1239. https://doi.org/10.1016/j.jaad.2020.05.143

Taylor, D. J., Hobby, A. E., Binns, A. M., & Crabb, D. P. (2016). How does age-related macular degeneration affect real-world visual ability and quality of life? A systematic review. *BMJ Open*, *6*(12). https://doi.org/10.1136/bmjopen-2016-011504

van Andel, E. M., Koopmann, B. D. M., Crouwel, F., Noomen, C. G., de Boer, N. K. H., van Asseldonk, D. P., & Mokkink, L. B. (2020). Systematic Review of Development and Content Validity of Patient-reported Outcome Measures in Inflammatory Bowel Disease: Do We Measure What We Measure? *Journal of Crohn's and Colitis*, *14*(9), 1299-1315. https://doi.org/10.1093/ecco-jcc/jjaa057

van der Have, M., van der Aalst, K. S., Kaptein, A. A., Leenders, M., Siersema, P. D., Oldenburg, B., & Fidder, H. H. (2014). Determinants of health-related quality of life in Crohn's disease: A systematic review and meta-analysis. *Journal of Crohn's and Colitis*, *8*(2), 93-106. https://doi.org/10.1016/j.crohns.2013.04.007

van Egdom, L. S. E., Oemrawsingh, A., Verweij, L. M., Lingsma, H. F., Koppert, L. B., Verhoef, C., Klazinga, N. S., & Hazelzet, J. A. (2019). Implementing Patient-Reported Outcome Measures in Clinical Breast Cancer Care: A Systematic Review. *Value in Health*, *22*(10), 1197-1226. https://doi.org/10.1016/j.jval.2019.04.1927

Van Hemelrijck, M., Sparano, F., Josephs, D., Sprangers, M., Cottone, F., & Efficace, F. (2019). Patient-reported outcomes in randomised clinical trials of bladder cancer: an updated systematic review. *BMC Urology*, *19*(1). https://doi.org/10.1186/s12894-019-0518-9

Van Hemelrijck, M., Sparano, F., Moris, L., Beyer, K., Cottone, F., Sprangers, M., & Efficace, F. (2020). Harnessing the patient voice in prostate cancer research: Systematic review on the use of patient‐reported outcomes in randomized controlled trials to support clinical decision‐making. *Cancer Medicine*, *9*(12), 4039-4058. https://doi.org/10.1002/cam4.3018

van Roij, J., Fransen, H., van de Poll-Franse, L., Zijlstra, M., & Raijmakers, N. (2018). Measuring health-related quality of life in patients with advanced cancer: a systematic review of self-administered measurement instruments. *Quality of Life Research*, *27*(8), 1937-1955. https://doi.org/10.1007/s11136-018-1809-4

Verberne, W. R., Das-Gupta, Z., Allegretti, A. S., Bart, H. A. J., van Biesen, W., García-García, G., Gibbons, E., Parra, E., Hemmelder, M. H., Jager, K. J., Ketteler, M., Roberts, C., Al Rohani, M., Salt, M. J., Stopper, A., Terkivatan, T., Tuttle, K. R., Yang, C.-W., Wheeler, D. C., & Bos, W. J. W. (2019). Development of an International Standard Set of Value-Based Outcome Measures for Patients With Chronic Kidney Disease: A Report of the International Consortium for Health Outcomes Measurement (ICHOM) CKD Working Group. *American Journal of Kidney Diseases*, *73*(3), 372-384. https://doi.org/10.1053/j.ajkd.2018.10.007

Vieta, A., Badia, X., Álvarez, E., & Sacristán, J. A. (2012). Which Nontraditional Outcomes Should Be Measured in Healthcare Decision-Making in Schizophrenia? A Systematic Review. *Perspectives in Psychiatric Care*, *48*(4), 198-207. https://doi.org/10.1111/j.1744-6163.2011.00325.x

Vos, J. A. M., Wieldraaijer, T., van Weert, H. C. P. M., & van Asselt, K. M. (2020). Survivorship care for cancer patients in primary versus secondary care: a systematic review. *Journal of Cancer Survivorship*, *15*(1), 66-76. https://doi.org/10.1007/s11764-020-00911-w

Wickwar, S., McBain, H. B., Ezra, D. G., Hirani, S. P., Rose, G. E., & Newman, S. P. (2014). What Are the Psychosocial Outcomes of Treatment for Thyroid Eye Disease? A Systematic Review. *Thyroid*, *24*(9), 1407-1418. https://doi.org/10.1089/thy.2014.0037

Wilson, M. K., Friedlander, M. L., Joly, F., & Oza, A. M. (2018). A Systematic Review of Health-Related Quality of Life Reporting in Ovarian Cancer Phase III Clinical Trials: Room to Improve. *The Oncologist*, *23*(2), 203-213. https://doi.org/10.1634/theoncologist.2017-0297

Yi, E., Ahuja, A., Rajput, T., George, A. T., & Park, Y. (2020). Clinical, Economic, and Humanistic Burden Associated With Delayed Diagnosis of Axial Spondyloarthritis: A Systematic Review. *Rheumatology and Therapy*, *7*(1), 65-87. https://doi.org/10.1007/s40744-020-00194-8

Zhao, Y., Brettle, A., & Qiu, L. (2018). The Effectiveness of Shared Care in Cancer Survivors—A Systematic

Review. *International Journal of Integrated Care*, *18*(4). https://doi.org/10.5334/ijic.3954
